# A novel yeast-derived aldehyde-reducing compound MF001 protects against alcohol-induced liver damage

**DOI:** 10.1371/journal.pone.0327648

**Published:** 2025-07-10

**Authors:** Eun-Ho Lee, Min-Hee Seo, Soo-Young Park, Sulagna Mukherjee, Jae-Ho Lee, Sora Kang, Ji-Yu Lee, Namgyu Lee, Hung Taeck Kwon, Seung-Soon Im

**Affiliations:** 1 Department of Physiology, Keimyung University School of Medicine, Daegu, Republic of Korea,; 2 Department of Physiology, College of Medicine and Institute of Medical Sciences, Gyeongsang National University, Jinju, Korea; 3 Picoentech Co., Ltd., Gyeong gi-do, Republic of Korea; 4 Department of Biomedical Science & Systems Biology, Dankook University, Cheonan, Republic of Korea; Université Clermont Auvergne - Faculté de Biologie, FRANCE

## Abstract

Alcohol-induced fatty liver disease is a significant contributor to global mortality, primarily resulting from excessive alcohol consumption and subsequent hepatic damage. This study investigated the therapeutic potential of MF001, an aldehyde-reducing compound derived from the yeast *Saccharomyces cerevisiae* in alcohol-induced liver damage. Using a Lieber-DeCarli ethanol diet-induced live disease model, we assessed the effects of MF001 on lipogenesis, oxidative stress, and inflammation. MF001 treatment significantly reduced lipid accumulation, as indicated by decreased expression of lipogenic genes. Moreover, MF001 suppresses reactive oxygen species (ROS) production indicated by reduced malondialdehyde levels and ROS-associated inflammatory markers, including Tnf-α, Il-6, and Mcp-1. Histological analysis revealed decreased hepatic lipid deposition and inflammation following MF001 administration. Furthermore, MF001 modulated alcohol metabolism by downregulating Cyp2e1 and Adh1, thereby decreasing acetaldehyde accumulation and improving liver function, as evidenced by normalized ALT and AST levels. Our findings suggest that MF001 alleviates alcohol-induced liver damage through its anti-inflammatory, antioxidant, and lipid-lowering properties, highlighting its potential as a function agent for preventing and treating alcohol-induced fatty liver disease.

## Introduction

At present, liver disease is the most prevalent cause of mortality annually, accounting for up to 4% of the global mortality. The primary causes of liver disease include alcohol consumption, viral hepatitis, and nonalcoholic fatty liver disease [1]. The risk of mortality from liver disease increases in proportion to alcohol intake, with a 3.2-fold increase in cardiovascular disease and a 5.1-fold increase in cancer due to liver cirrhosis [2]. Accumulation of fat in the liver of individuals who consume alcohol over an extended period is the underlying cause of fatty liver [3]. The histological appearance of fat droplets is characterized by their deposition in the cytoplasm of hepatocytes, with a predilection for regions surrounding the central vein, extending toward the center of the hepatic lobule and ultimately reaching the portal area [4].

Among the globally leading liver diseases related to heavy alcohol consumption is alcohol-associated liver disease (ALD) [5], resulting in haptic inflammation due to response produced by various cytokines such as TNF-α, IFN-γ, and interleukin family proteins in the liver by the Kupffer cells. In a contrasting and protective phenomenon liver secretes anti-inflammatory interleukins like IL-6 and IL-10 by activating the STAT3 in Kupffer cells to inhibit the alcoholic liver inflammation [6]. Sustained alcohol consumption results in fat accumulation in liver cells through mechanisms that disrupt lipid metabolism, leading to alcoholic fatty liver. Alcohol consumption alters the NAD + -NADH balance, reducing mitochondrial fatty acid breakdown and increasing fat synthesis. It also increases the expression of SREBP-1c, which regulates lipid-producing genes, while decreasing PPARα expression, which supports fatty acid oxidation. These changes are due to reduced AMPK activity, a key energy metabolism regulator, accompanied by increased ER stress and ROS accumulation. Together, these factors contribute to the development of alcoholic fatty liver [7].

Several studies have demonstrated that excessive alcohol consumption results in the development of hangovers, which are typified by a constellation of unpleasant mental and physical symptoms, including headache, nausea, vomiting, fatigue, and muscle pain [8,9]. Other severe symptoms of hangovers include neurocognitive impairments that affect executive function, resulting in deficits in attention, memory, and psychomotor skills [10]. The primary causes of hangovers can be attributed to the toxicity of alcohol and its metabolites, damage to liver tissue resulting from the production of reactive oxygen species (ROS), and increased concentration of acetaldehyde in the bloodstream [11]. Approximately 25% of alcohol is absorbed by the stomach within 30 min; however, when consumed on an empty stomach, more than 90% is absorbed from the small intestine within two hours [12]. The absorbed alcohol is oxidized to acetaldehyde through the catalytic reaction of alcohol dehydrogenase (ADH) within liver cells and then to non-toxic acetic acid by acetaldehyde dehydrogenase (ALDH) [13]. This transformation of alcohol into acetaldehyde and then into acetate is facilitated by ALDH [14]. The metabolism of ethanol (EtOH) by ADH and ALDH also influences drinking behavior. Modifying ALDH metabolism through interventions could be an effective strategy for reducing the incidence of diseases associated with EtOH consumption [15]. A recent study demonstrated that liver-targeted ALDH2 inhibition is a potential treatment for alcohol use disorders [16]. Furthermore, considering alternative strategies for pain relief following alcohol consumption and for the reduction of hangovers is imperative.

Currently, the use of an aldehyde-reducing composition (ARC) as an extract lysate is regarded as a potential solution for recovering from alcohol-induced hangovers. This ARC compound was developed from a novel yeast strain, designated “Kwon P-1,2,3,” which was generated from both ALDH and glutathione (GSH) with high efficiency. The strain was optimized using a fermentation process that used chemical mutagenesis instead of genetic recombination. It was selected for GSH and ALDH production in the presence of methylglyoxal and lysine. The mutant strain is safe for use in food, feed, cosmetics, and medicines, and is generally recognized as safe (GRAS) [17–19]. A recent study demonstrated that ARC reduced acetaldehyde and malondialdehyde levels in alcohol-administered animal models with high ALDH activity and anti-aldehyde and antioxidant properties [20]. Earlier studies demonstrated that oral intake of ARC-related compound powder elevated the metabolic potential of ALDH2 while lowering serum acetaldehyde levels *in vivo* models [21]. In light of these findings, ARC is currently being marketed in Korea as a means of alleviating the symptoms of alcohol hangovers, thus demonstrating its safety, even in humans [17]. Nevertheless, the impact of ARC on excessive alcohol consumption remains unclear. This study was designed to evaluate the hypothesis that the ARC-related compound ALD-gluta-yeast powder (MF001) can protect against alcohol-induced fatty liver disease and reduce acetaldehyde degradation in the livers of mice fed a Lieber-DeCarli (LD) EtOH diet.

## Materials and methods

### 1.1. Preparation of MF001 compounds

MF001 compounds were derived from the GRAS strain of *Saccharomyces cerevisiae* and comprise proprietary, differentiated, and patented rights. The compounds were prepared and standardized by PICO Entech Co. Ltd. (Seongnam, Korea) as previously described [20]. In summary, MF001 yeast was cultivated in an incubator using a yeast extract peptone dextrose medium comprising 1% yeast extract, 2% glucose, and 1% peptone for 24 h. This was followed by fermentation for 48 h in a 5 L fermenter (Marado-05D-PS, CNS, Daejeon, Korea). Yeast pellets were obtained via centrifugation at 2,000 × *g* for 10 min in a high-speed centrifuge (Supra R22; Hanil Scientific Inc., Gimpo, Korea) and then frozen for 2 d in a cryogenic freezer (CLN-52U; Nihon Freezer, Tokyo, Japan). The frozen yeast pellet was subjected to freeze-drying (FDU-7006, Operon, Gimpo, Korea) to obtain yeast extract powder. Subsequently, the yeast extract powder (1 g) was dissolved in 10 mL phosphate-buffered saline (PBS, pH 8.0) containing the Xpert protease inhibitor cocktail solution (P3100, Thermo Fisher Scientific, Lenexa, KS, USA) and subjected to homogenization using steel beads with a bead homogenizer (Taco™ Prep Bead Beater, GeneReach, Taichung, Taiwan). The homogenized extract was subjected to another round of homogenization using a sonicator (Bioruptor KRB-01, BMS, Tokyo, Japan) for 20 cycles, after which it was centrifuged to separate the supernatant. This latter fraction was filtered with a 0.2 μm filter (HYUNDAI MICRO Co. Ltd, Seoul, Korea) and subsequently used for research.

### 1.2. Animal model

C57BL/6J mice are the optimal strain for *ad libitum* EtOH feeding and exhibit a preference for alcohol consumption [22]. Wild-type (WT) mice were bred in a clean mouse-breeding facility and free from specific pathogens. Ten-week-old male mice with body weights exceeding 25 g were used for chronic binge-feeding experiments, with six mice per group. The LD EtOH diet-induced alcoholic fatty-liver model was prepared in accordance with the guidelines set forth in a previously described NIAAA model [22]. Two sets of experiments were conducted to assess the effects of MF001. The initial phase of the study involved the development of an alcohol-induced fatty liver metabolic dysfunction model. This was achieved by feeding mice an LD EtOH diet for 5 d, followed by the administration of either MF001 (with varied doses, 0.1, 0.2, and 2 g/kg) or the reference drug, *Hovenia dulcis* Thunb. extract powder (HDT) of 0.5g/kg, for 0−3 weeks. In the second set of experiments, administration of MF001 or HDT was initiated following a 3-week period of LD EtOH diet consumption (n = 6 per group). In both experiments, prior to euthanasia, the mice were administered an oral gavage of EtOH, whereas the vehicle group received an oral gavage of maltose. During this experiment to minimize animal suffering, animals were closely monitored for signs of pain, distress, or illness. When necessary, analgesics (Buprenorphine and carprofen) at appropriate doses were administered to alleviate discomfort, following IACUC recommendations. Animals exhibiting significant weight loss, abnormal behavior, or severe clinical signs were promptly euthanized. At the endpoint of the study or when euthanasia was required, animals were sacrificed by CO_2_ inhalation followed by cervical dislocation to ensure death. All animal experiments were conducted in accordance with the guidelines of the Laboratory Animal Ethics Committee of Keimyung University College of Medicine (KM-2023-10).

### 1.3. Mouse primary hepatocyte isolation

Hepatocytes were isolated from the livers of mice using the perfusion method, as previously described [23]. Mouse primary hepatocytes were isolated from 10-week-old male WT mice via liberase perfusion. In a tissue culture hood, the minced liver capsule was removed using forceps and shaken gently until the hepatocytes were released. Subsequently, the cells were filtered through a 100 µm nylon cell strainer, and the filtrate was subjected to centrifugation at 50 × *g* for 1 min at 4 °C. The hepatocyte suspension was plated on rat-tail collagen type I-coated plates in an attachment medium (William’s E medium supplemented with 10% fetal bovine serum (FBS), 1% GlutaMax, and 1% P/S). Following a 4-hour period during which the cells were allowed to attach, they were washed and incubated in high-glucose DMEM supplemented with 10% FBS and 1% P/S.

### 1.4. Preparation of palmitic acid (PA) solutions

Bovine serum albumin (BSA; A3803, Sigma-Aldrich, St. Louis, MO, USA)-conjugated PA (P0500; Sigma-Aldrich) was prepared using a modified version of a previously described method [24]. Initially, 150 mM PA was dissolved in 50% EtOH for 15 min and 10% BSA was dissolved in serum-free DMEM. The solutions were then mixed and dissolved to a concentration of 7.5 mM, and then filtered through a 0.2 µm filter. The final concentration of PA conjugated with BSA was determined by further dilution to 200 μM.

### 1.5. Flow cytometry analysis

Primary hepatocytes obtained from WT mice were divided into three groups and treated with palmitate (0.5 mM), EtOH (100 mM), or MF001. Following a 24-hour incubation period, 10 mM carboxy-2′,7′-dichlorodihydrofluorescein diacetate (carboxy-H2DCFDA; Molecular Probes, Eugene, OR, USA) was added to the cells, which were then incubated at 37 °C for 30 min. Cells were harvested by treatment with 0.05% trypsin and washed twice with cold PBS. Cells that had been conjugated with carboxy-H2DCFDA-FITC (excitation, 494 nm; emission, 524 nm) were detected using the FL1 setting of the FACS Calibur (BD Biosciences, San Jose, CA, USA).

### 1.6. Oil Red O (ORO) staining

Primary hepatocytes were isolated from control (Con) mice, PA-conjugated EtOH-treated mice, and PA-conjugated EtOH-treated mice treated with MF001. The cells were then cultured in 100 mm culture dishes until they reached 100% attachment and confluency. Subsequently, the cells were washed with PBS and fixed with 4% paraformaldehyde (pc2031-100-00; Biosesang, Seongnam, Korea) for 30 min at room temperature, followed by two washes with PBS. A mixture of ORO solution (0.6% ORO dye in isopropanol) and water (6:4 ratio) was applied to the cells for 20 min, after which the cells were washed five times with deionized water. Subsequently, the cells were covered with water and observed under a microscope. The intracellular lipid droplet content was quantified at an absorbance of 492 nm using the standard expression for lipid droplets.

### 1.7. Histological staining

Liver specimens were fixed in 4% paraformaldehyde, embedded in paraffin, and cut into 4 μm-thick sections. The sections were dewaxed, hydrated, and stained in accordance with the established protocols, utilizing hematoxylin and eosin (BBC Biochemical, Mount Vernon, WA, USA) and F4/80 (70076, CST, Danvers, MA, USA).

### 1.8. Hepatic triglyceride (TG) and total cholesterol (TC) measurements

To extract lipids from liver tissue, 50 mg of liver tissue was collected and homogenized with chloroform and methanol (2:1 ratio) in a glass tube. The resulting mixture was dried under nitrogen gas. Subsequently, a solution comprising chloroform and methanol in a 1:1 ratio, along with 50 mM lithium chloride (LiCl), was prepared and centrifuged at 500 × *g* for 10 min. The separated lower layer was then collected and the collected solution was dried with nitrogen gas. The dried sample was then resuspended in 10 mM LiCl, chloroform, and methanol, and centrifuged to collect the lower layer. This process was repeated to obtain the dried samples. Following the addition of chloroform to the dried sample, absorbance was measured at 550 nm using a TG or TC kit (Asan Pharm Co. Seoul, Korea) and Infinite 200 PRO (Tecan Trading AG, Männedorf, Switzerland).

### 1.9. RNA isolation and real-time quantitative polymerase chain reaction (qPCR)

Total RNA was extracted from cells and mouse livers using TRIzol reagent (Invitrogen) and reverse-transcribed using a cDNA iScript kit (Bio-Rad, Hercules, CA, USA). Quantitative reverse transcription-polymerase chain reaction (qPCR) was performed using a CFX96 Bio-Rad qPCR system (Bio-Rad). Polymerase chain reactions were conducted using iQ™ SYBR Green Supermix (Bio-Rad) and primers specific to the target sequences (S1 Table in S1 File). The mRNA levels were normalized to the expression in L32 (Con) cells and calculated using the comparative threshold cycle (C_T) method. All samples were analyzed in triplicate and the mean values were calculated. The qPCR amplification was performed 39 times. The cycling parameters were 95 °C for 3 min, 95 °C for 10 s, and 55 °C for 30 s. Subsequently, a melting curve was generated by increasing the temperature by 0.5 °C every 5 s to 95 °C.

### 1.10. Western blot analysis

Proteins were isolated from cells and mouse livers according to a modified protocol previously established by [25]. For protein extraction, cells and frozen livers were homogenized in RIPA buffer and T-PER lysis buffer (Thermo Fisher Scientific, Wilmington, MA, USA) with a protease and phosphatase inhibitor cocktail (Thermo Fisher Scientific). The protein concentration of the samples was determined by measuring the absorbance at 562 nm using a bicinchoninic acid assay kit (Thermo Fisher Scientific). The sample was then separated using electrophoresis on a 10% sodium dodecyl sulfate-polyacrylamide gel and transferred to a 0.2 mm nitrocellulose membrane for quantification (GE Healthcare, Amersham, Chicago, IL, USA). Subsequently, membranes were incubated in 5% skim milk/TBST (20 mM Tris-HCl, 137 mM NaCl, and 0.1% Tween 20; pH 7.4) at room temperature for 1 h. Subsequently, primary antibodies for fatty acid synthetase (FASN) (3180, Cell Signaling Technology, Danvers, MA, USA) and acetyl-CoA carboxylase 1 (ACC1) (4190, Cell Signaling Technology) were used. Stearoyl-CoA desaturase 1 (SCD1) (2794, Cell Signaling Technology) and β-actin (A5441, Sigma-Aldrich) were diluted in 3% BSA (VWR, Avantor, PA, USA)/TBST and incubated at 4 °C for 16 h. The secondary antibodies, anti-mouse and anti-rabbit (Santa Cruz Biotechnology), were diluted in 5% skimmed milk/TBST. Following a 1-hour incubation period, protein bands were detected using ECL (Bio-Rad) (S1 Data in S1 File), and chemiluminescence was analyzed using a Fusion Fx imaging system (Vilber Lourmat, Collégien, France).

### 1.11. Enzyme-linked immunosorbent assay

Serum TG and TC were extracted using the methods described by [26] and [27], respectively, as previously outlined. Serum TG levels were quantified using a TG assay kit (AM 157S-K, Asan Pharm Co.), whereas serum TC levels were quantified using a TC assay kit (AM 202-K, Asan Pharm Co.). The absorbance of the samples and standard was measured at 500 or 550 nm using an Infinite 200 PRO (Tecan Trading AG). Hepatic injury was determined by measuring the serum levels of alanine aminotransferase (ALT) and aspartate aminotransferase (AST) using spectrophotometric assay kits (AM 102-K and AM 103-K; Asan Pharm Co.). The absorbance values of serum biochemicals were measured at a wavelength of 505 nm. The concentration of non-esterified fatty acids (NEFAs) in serum was determined using a LabAssay™ NEFA Kit (633−52001, FUJIFILM, Osaka, Japan), following the manufacturer’s instructions. Acetaldehyde concentration from mouse serum and primary hepatocyte cultured media was determined using an acetaldehyde UV-method kit (10668613035, R-Biopharm AG, Darmstadt, Germany) according to the manufacturer’s instructions. The concentration of MDA, a lipid peroxidation product, in the cell lysates was determined using a lipid peroxidation assay kit (ab118970, Abcam, Cambridge, UK) in accordance with the manufacturer’s instructions. The level of liver detoxification in serum was evaluated using a γ-glutamyl transferase (GGT/γ-GTP) activity assay kit (E-BC-K126-M, Elabscience Bionovation Inc., Houston, TX, USA) according to the manufacturer’s instructions. The cell culture supernatants were centrifuged for 10 min at 12,000 rpm and 4 °C. The absorbance was measured and analyzed using an Infinite 200 PRO spectrophotometer (Tecan Trading AG). The level of cell supernatant and mouse serum was evaluated using a mouse ALDH2 ELISA kit (A314400, Antibodies, Cambridge, UK), mouse IL-1 beta/IL-1F2 ELISA kit (MLB00C, R&D Systems, Minneapolis, MN, USA), and mouse TNF-alpha ELISA kit (MTA00B, R&D Systems) according to the manufacturer’s instructions.

### 1.12. Statistical analysis

The results obtained in this study were analyzed using GraphPad Prism 9.5.1 (GraphPad Software Inc., California, USA), and statistical significance was determined using an independent sample multiple t-tests or one-way ANOVA (S2 Data in S1 File). Statistical significance was determined using the following criteria: * *p <* 0.05, ** *p <* 0.01, *** *p <* 0.001, # *p <* 0.05, ## *p <* 0.01, and ### *p <* 0.001.

## Results

### 2.1. MF001 reduces lipogenesis and inflammation in primary hepatocytes

**Fig 1:**
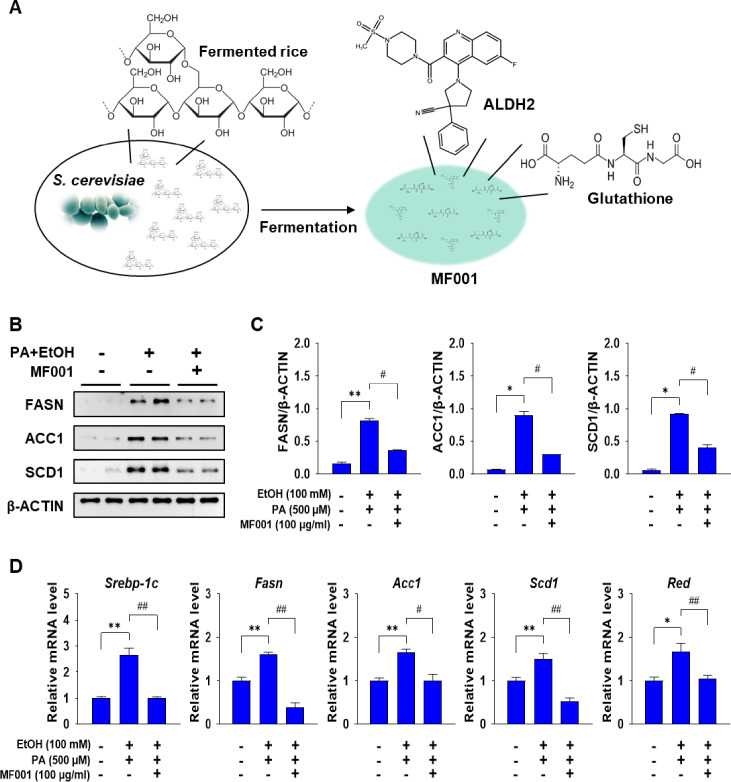
MF001 reduces the expression of lipogenic genes and proteins. (A) Schematic representation of MF001. (B) FASN, ACC1, and SCD1 protein levels in mouse primary hepatocytes determined using western blotting. (C) Quantification via densitometric analysis using β-ACTIN for normalization. (D) Lipogenic gene expression levels of *Srebp-1c*, *Fasn*, *Acc1*, *Scd1,* and *Red* determined using qPCR in EtOH-, PA-, and MF001-treated mouse primary hepatocytes. Values represent the mean ± SEM. ^*^p < 0.05, and ^**^p < 0.01 compared to mock primary hepatocytes. ^#^*p* < 0.05, and ^##^*p* < 0.01 compared with EtOH- and PA-treated mouse primary hepatocytes.

**Fig 2:**
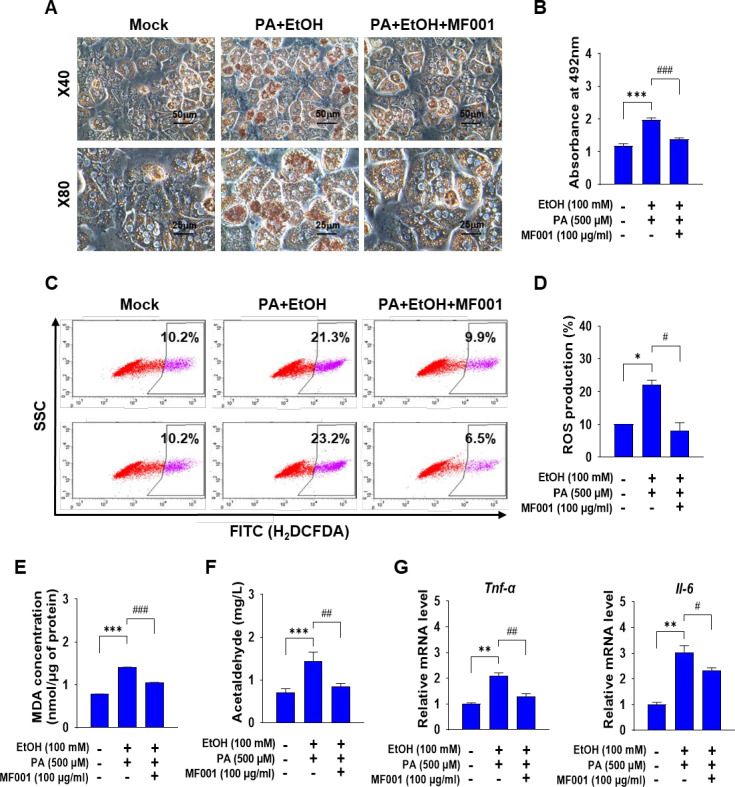
MF001 alleviates lipid accumulation in primary hepatocytes. (A) Representative microscopic images of ORO staining (scale bars: 50 and 25 μm) and (B) quantification of ORO-positive staining in mouse primary hepatocytes (n = 3 per group). (C) Quantification of ROS using H_2_DCFDA in FACS analysis and (D) percentage ROS production between the various groups in mouse primary hepatocytes. (E) Analysis of MDA concentrations in mouse primary hepatocytes. (F) Determination of acetaldehyde levels from supernatants of primary hepatocytes. (G) Expression levels of inflammatory genes *Tnf-α* and *Il-6* in mouse primary hepatocytes. Values represent the mean ± SEM. ^*^p < 0.05, ^**^p < 0.01, and ^***^p < 0.001 compared to mock primary hepatocytes. ^#^*p* < 0.05, ^##^*p* < 0.01, and ^###^*p* < 0.001 compared to EtOH- and PA-treated primary hepatocytes.

The ALDH-related compound, designated MF001, exhibits ARC activity and was derived from a novel strain of yeast (Fig 1A). The initial objective was to ascertain the effect of MF001 on alcohol-induced fatty liver cells. Primary hepatocytes were isolated from a normal mouse liver and treated with 100 mM EtOH in conjunction with 500 µM PA to induce excess lipid production in liver cells. This was followed by treatment with 100 µg/mL of MF001. A notable decline in the expression of proteins associated with lipogenesis was observed in the liver, including FASN, ACC1, and SCD1, which were elevated in the presence of EtOH and PA (Fig 1B and 1C). A comparable pattern of diminished lipogenic gene expression (*sterol-regulatory element binding protein-1c* (*Srebp-1c*), *Fasn*, *Acc1*, *Scd1*, and *HMG-CoA reductase* (*Red*)) was evident following MF001 treatment (Fig 1D). Moreover, the accumulation of lipids in primary hepatocytes was confirmed via ORO staining. Microscopic images and subsequent quantification demonstrated a notable accumulation of lipid droplets in the PA + EtOH group when compared to the Con group, accompanied by a striking reduction in lipid content in the cells treated with MF001 (Fig 2A and 2B). These findings suggested that MF001 has the potential to reduce lipogenesis and lipid accumulation in hepatocytes. Excessive accumulation of lipids resulting from EtOH exposure can result in the formation of ROS and the subsequent onset of hepatic inflammation. ROS levels were quantified using FACS analysis to ascertain the ROS-scavenging capabilities of MF001. As anticipated, the PA + EtOH group demonstrated a considerable elevation in ROS production, reaching 21.3% and 23.2% in comparison to Con cells (10.2%). Conversely, following MF001 treatment, notable reductions in ROS levels were observed, reaching 9.9% and 6.5%, respectively (Fig 2C and 2D). Furthermore, the impact of MF001 on lipid peroxidation was confirmed via the MDA assay, which demonstrated a decline in MDA activity following treatment with MF001 (Fig 2E). Treatment with MF001 at a concentration of 100 µg/ml also showed significant reduction in the acetaldehyde levels of primary hepatocytes (Fig 2F) compared to the ethanol treated group. To ascertain whether the inflammatory response was regulated by lipid peroxidation, the gene expression of inflammatory cytokines, namely tumor necrosis factor-α (*Tnf-α*) and *interleukin*-6 (*Il-6*), was quantified. The results demonstrated a decline in expression levels comparable to those observed in the context of lipid peroxidation (Fig 2G). These findings suggest that MF001 exerts anti-inflammatory effects by modulating ROS generation and lipid peroxidation.

### 2.2. MF001 protects against alcohol-induced fatty liver symptoms

**Fig 3:**
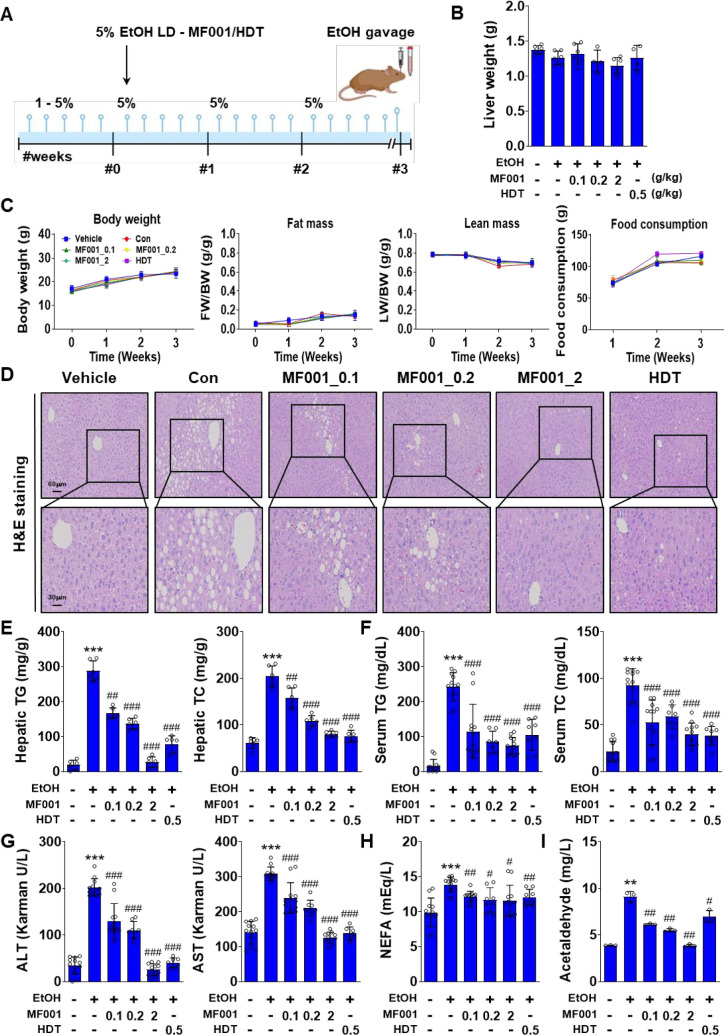
MF001 ameliorated alcohol-induced fatty liver phenotypes in mice. (A) Schematic representation of LD EtOH diet in mice for 3 weeks (n = 6 per group). (B) Quantification of liver weight. (C) Body weight, fat mass, lean mass, and food consumption between the various groups of mice models comprising Vehicle (negative Con), Con (treated with EtOH), the reference drug (HDT), and different doses of MF001 (0.1, 0.2, and 2 g/kg). (D) Histological analysis of liver sections using H&E staining to determine lipid accumulation in mouse liver tissues (scale bars: 60 μm and 30 μm). Measurement of (E) hepatic TG and TC, (F) serum TG and TC, (G) serum ALT and AST, (H) serum NEFA, and (I) serum acetaldehyde concentrations in the different groups of mice treated with or without MF001. Values represent the mean ± SEM. ^**^p < 0.01, and ^***^p < 0.001 com*p*ared to vehicle WT mice. ^#^*p* < 0.05, ^##^*p* < 0.01, and ^###^*p* < 0.001 com*p*ared to LD EtOH diet WT mice.

To understand the protective effect of MF001 in alcohol induced fatty-liver disease, mice models were used. Initially, the mice were fed with LD EtOH diet (1−5%) for 5days followed by random assignment to one of several groups and treated with different doses of MF001 (0.1, 0.2, or 2 g/kg). The Con group of mice that did not consume alcohol was designated as the vehicle group, whereas the group that did not receive compound treatment was designated as the Con group. The reference drug used for recovery from fatty liver disease was designated as the HDT group. The LD EtOH diet was administered to mice for a period of three weeks, as illustrated in Fig 3A, with the objective of inducing alcohol-induced fatty liver syndrome. Prior to euthanasia, the mice administered an oral gavage of EtOH, whereas the vehicle group received an oral gavage of maltose. Group Con: HDT was used as the reference drug for fatty liver recovery. Although there was no significant difference in the liver weight between the groups (Fig 3B), we observed slight elevations in whole-body weight and food consumption in all groups with ascending time (from week 0 to week 3), no significant increase in fat mass, and reduction in lean mass (Fig 3C). Histological staining of liver tissues by H&E stain depicted clearly the reduction of fat accumulation in the MF001 treated mice groups in comparison to ethanol treated control, indicating reduced fat deposition after consumption of MF001 by the mice (Fig 3D). Subsequently, other biochemical parameters, including hepatic TG, serum TG, hepatic TC, and serum TC levels, were evaluated. A significant increase was observed in the LD EtOH diet-induced Con mice group compared to the vehicle group, whereas MF001, particularly at a dose of 2 g/kg, demonstrated the ability to attenuate TG and TC accumulation (Fig 3E and 3F). Serum ALT and AST levels, indicators of hepatotoxicity, were reduced to within the normal range after treatment with 2 g/kg MF001 (Fig 3G). The serum NEFA level exhibited a 1.5-fold increase in all groups relative to the vehicle group, indicative of EtOH-induced fatty liver induction. However, a slight reduction was observed after treatment with MF001 and HDT (Fig 3H), suggesting that MF001 exerted a protective effect against alcohol-induced fatty liver. The level of acetaldehyde, a metabolite of EtOH, decreased to normal, which indicated the inhibition of hepatotoxicity by MF001 (Fig 3I). This process might regulate liver lipogenesis. Moreover, induction of MF001 in primary hepatocytes reduced the gene expressions of fatty acid oxidizing genes, *Pparα*, *Pgc-1α*, *Cpt-1α,* and *Cpt-1β* (S1A Fig in S1 File). Treatment with 100 µg/ml of MF001 also reduced the expressions *Cyp2e1*, *Adh1,* and *Aldh2*, genes regulating aldehyde and EtOH metabolism (S1B Fig in S1 File). In addition, MF001 treatment in primary hepatocytes also showed reduced levels of ALDH2 enzyme, suggesting the definitive role of MF001 in reducing fatty acid oxidation and ethanol metabolism.

### 2.3. MF001 attenuates lipid accumulation and peroxidation by reducing ROS formation

**Fig 4:**
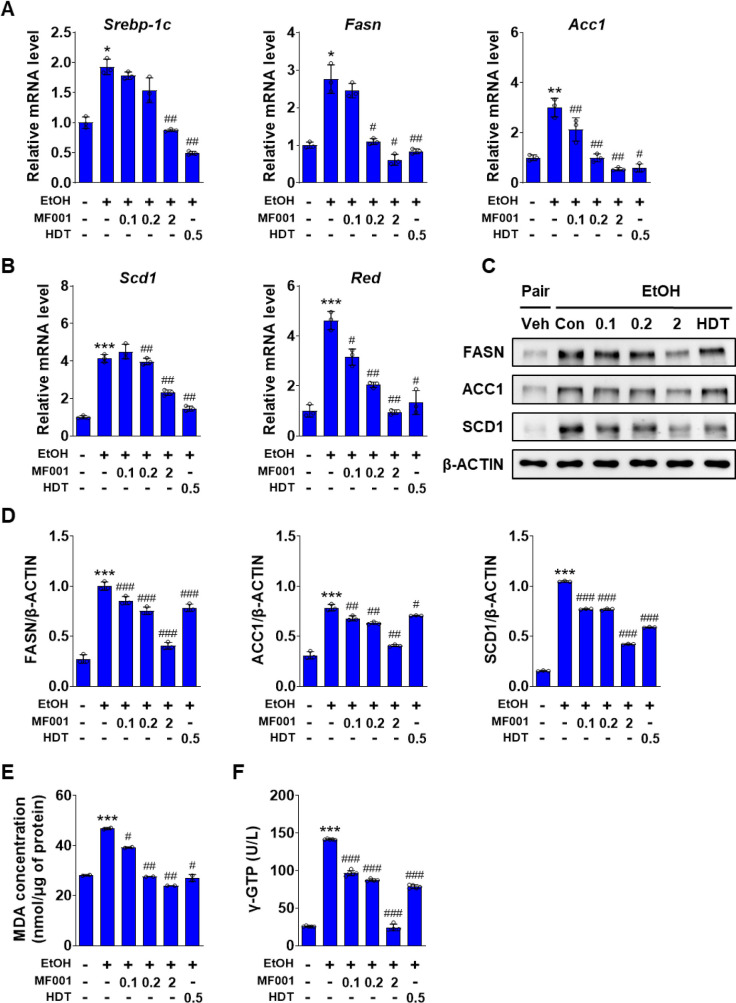
MF001 reduces lipid accumulation and peroxidation in liver tissues. Expression of major lipogenic genes, such as (A, B) *Srebp-1c*, *Fasn*, *Acc1*, *Scd1*, and *Red* was assessed using qPCR, and protein levels of FASN, ACC1, and SCD1 were determined using western blotting (C). (D) Protein expression was quantified using densitometric analysis, normalized to β-ACTIN, across various groups of mice models comprising Vehicle (negative Con), Con (treated with EtOH), the reference drug (HDT), and different doses of MF001 ranging between 0.1, 0.2, and 2 g/kg. (E) Quantification of lipid peroxidation by analyzing the levels of MDA, and (F) analysis of γ-GTP. Values represent the mean ± SEM. ^*^p < 0.05, ^**^p < 0.01, and ^***^p < 0.001 compared to vehicle WT mice. ^#^*p* < 0.05, ^##^*p* < 0.01, and ^###^*p* < 0.001 compared to LD EtOH diet WT mice.

**Fig 5:**
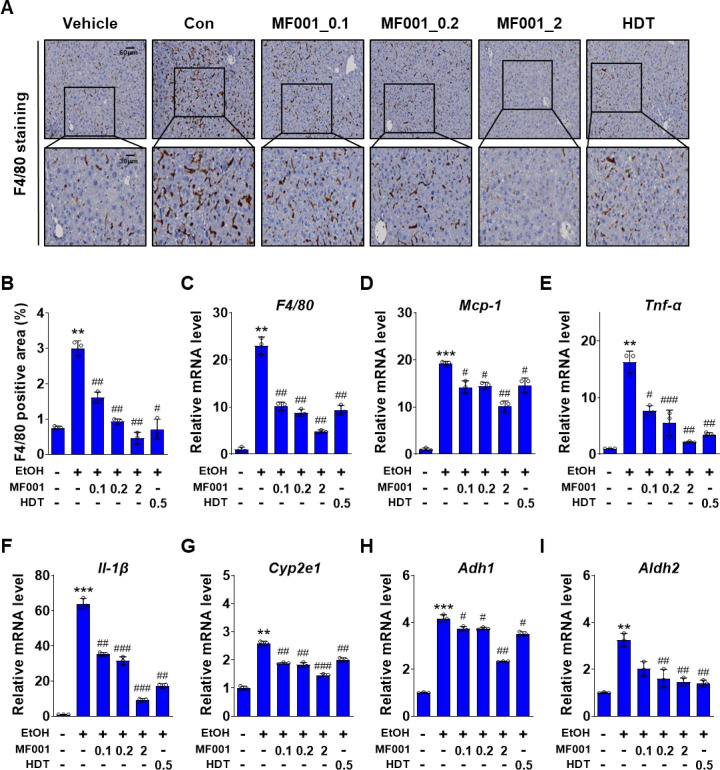
MF001 protects against alcohol-induced liver inflammation. (A) Histological analysis of hepatic macrophage infiltration by F4/80 staining from liver tissues (scale bars: 60, and 30 μm) and (B) its quantitative graphical representation. (C-F) Expression levels of inflammatory genes for *F4/80*, *Mcp-1*, *Tnf-α*, and *Il-1β* in mouse liver tissue. (G-I) *Cyp2e1*, *Adh1*, and *Aldh2* mRNA levels were measured using qPCR. Values represent the mean ± SEM. ^**^p < 0.01, and ^***^p < 0.001 compared to vehicle WT mice. ^#^*p* < 0.05, ^##^*p* < 0.01, and ^###^*p* < 0.001 compared to LD EtOH diet WT mice.

The role of MF001 in lipid accumulation and cellular membrane peroxidation was investigated using a mouse model of alcohol-induced fatty liver disease. To substantiate this hypothesis, the expression levels of the lipogenic genes were quantified. As illustrated in Fig 4A and 4B, the expression of *Srebp-1c*, which is associated with lipid metabolism, decreased. The expression of lipogenic genes, including *Fasn*, *Acc1*, *Scd1*, and *Red*, also decreased in a concentration-dependent manner following MF001 treatment. At elevated MF001 concentrations, the expression level of the lipogenic genes was maintained at an optimal level comparable to that observed in the vehicle without an LD EtOH diet. The protein levels of FASN, ACC1, and SCD1 were regulated by MF001 (Fig 4C and 4D). MF001 treatment reduced the genes involved in fatty acid oxidation, *Pparα*, *Pgc-1α*, *Cpt-1α*, and *Cpt-1β* in a dose dependent manner (S2A Fig in S1 File). The effect of MF001 on lipid peroxidation and liver detoxification caused by alcohol intake was confirmed by a decrease in these levels following MF001 treatment, which exhibited similar expression patterns to those of the lipogenic genes (Fig 4E and 4F). Thus, MF001 is effective in the treatment of alcohol-induced fatty liver disease. Alcoholic liver disease results in liver damage via sterile inflammation [28]. Therefore, we investigated the role of MF001 in alcohol-induced liver inflammation. Histological analysis revealed that F4/80 staining for inflammation was significantly elevated in the Con mice, whereas treatment with MF001 resulted in a notable reduction in this staining (Fig 5A and 5B). The expression of the *F4/80* gene exhibited a similar pattern (Fig 5C). Furthermore, the expression levels of inflammation-related genes, including *monocyte chemoattractant protein-1 (Mcp-1*), *Tnf-α*, and *Il-1β*, were also decreased by MF001 treatment (Fig 5D–5F). In addition, serum levels of TNF-α and IL-1β reduced upon treatment with MF001 (S2B Fig in S1 File). Cytochrome P450 family 2 subfamily E polypeptide 1 (CYP2E1) is responsible for EtOH metabolism, resulting in the generation of ROS and acetaldehyde. These byproducts have been linked to liver damage and toxicity in other organs [29]. ADH1 is the primary enzyme involved in EtOH metabolism within the liver [30]. The expression levels of *Cyp2e1* and *Adh1*, which are involved in acetaldehyde production and EtOH metabolism, significantly decreased after treatment with 2 g/kg MF001 (Fig 5G and 5H) and gene expression and serum level activity of ALDH2 was also decreased (Fig 5I and S2C Fig in S1 File). Thus, the inflammatory response associated with alcohol-related liver damage could be regulated by MF001.

Two sets of experiments were conducted to assess the effects of MF001. The initial phase of the study involved the development of an alcohol-induced fatty liver metabolic dysfunction model. This was achieved by feeding mice an LD EtOH diet for 5 d, followed by the administration of either MF001 (with varied doses, 0.1, 0.2, and 2 g/kg) or the reference drug, *Hovenia dulcis* Thunb. extract powder (HDT) of 0.5g/kg, for 0–3 weeks. In the second set of experiments, administration of MF001 or HDT was initiated following a 3-week period of LD EtOH diet consumption (n = 6 per group). In both experiments, prior to euthanasia, the mice were administered an oral gavage of EtOH, whereas the vehicle group received an oral gavage of maltose.

### 2.4. MF001 alleviates alcohol-induced liver inflammation and fatty liver symptoms in mice

**Fig 6:**
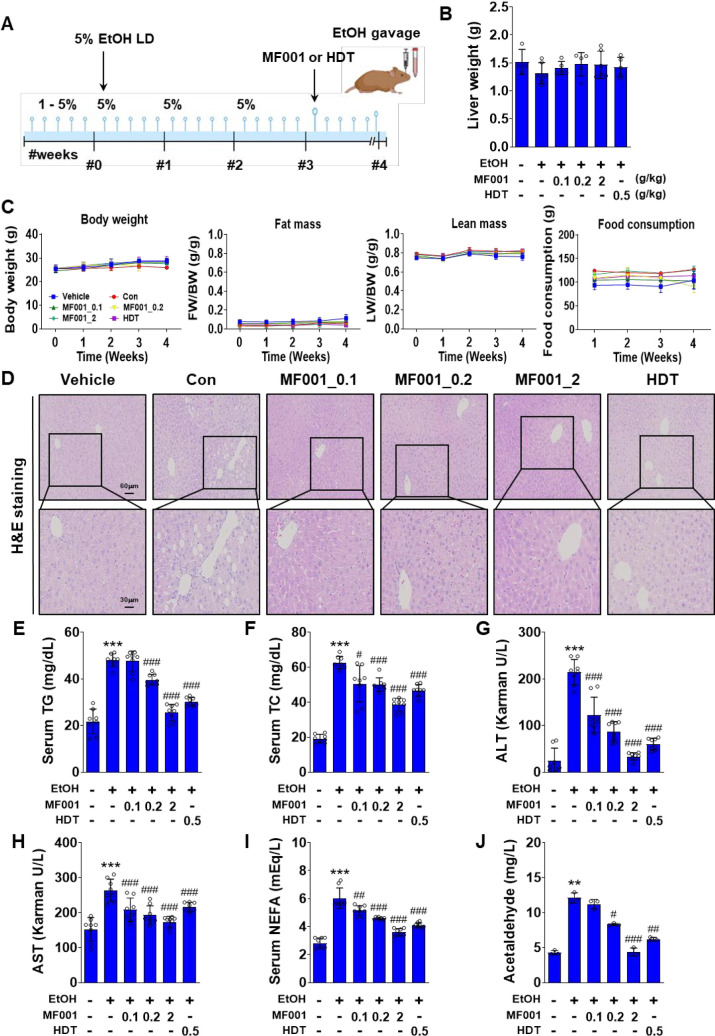
MF001 attenuates alcohol-induced fatty liver phenotypes in mice. (A) Schematic representation of the LD EtOH diet in mice for 4 weeks followed by administration of MF001 or HDT (n = 6 per group). (B) Quantification of liver weight. (C) Body weight, fat mass, lean mass, and food consumption were normalized to the body weight. (D) Histological analysis of liver sections using H&E staining to determine lipid accumulation in mouse liver tissues (scale bars: 60 μm and 30 μm). Measurement of (E, F) serum TG and TC, (G, H) serum ALT and AST, (I) serum NEFA, and (J) serum acetaldehyde concentrations in the different groups of mice treated with or without MF001. Values represent the mean ± SEM. ^**^p < 0.01, and ^***^p < 0.001 com*p*ared to vehicle WT mice. ^#^*p* < 0.05, ^##^*p* < 0.01, and ^###^*p* < 0.001 com*p*ared to LD EtOH diet WT mice.

**Fig 7:**
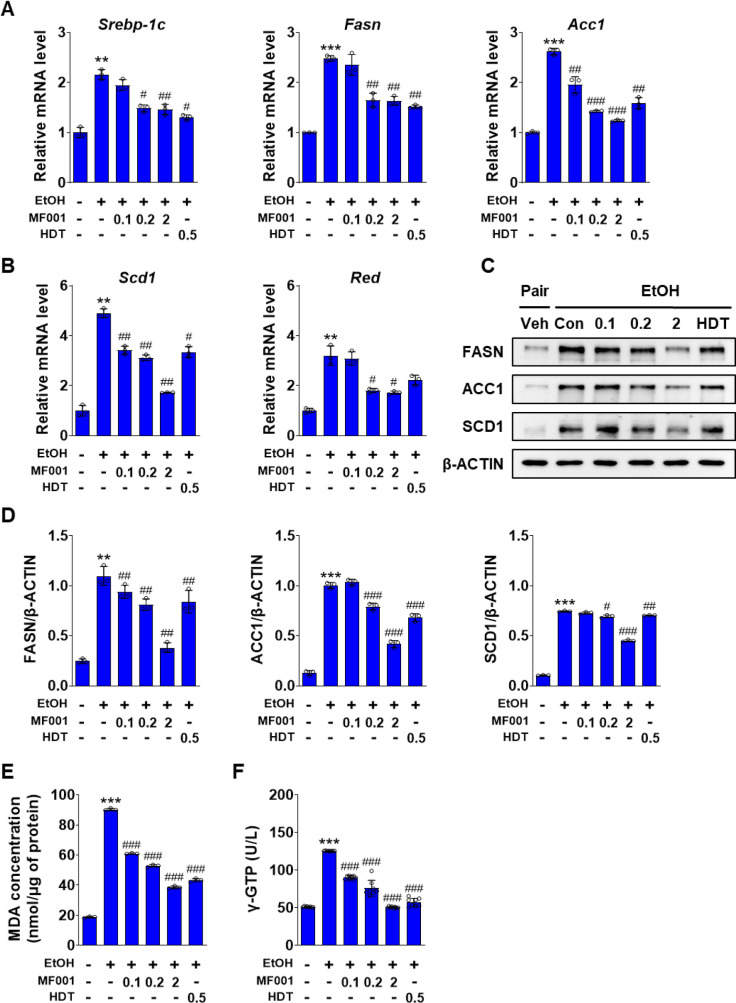
MF001 liberates alcohol-induced lipogenesis in mice. (A, B) Lipogenic gene expression levels for *Srebp-1c*, *Fasn*, *Acc1*, *Scd1*, and *Red* in livers of LD EtOH diet WT mice. (C, D) Western blot analysis and quantification of FASN, ACC1, and SCD1 protein levels in the livers of LD EtOH diet WT mice. (E, F) Analysis of MDA and γ-GTP concentrations in serum. Values represent the mean ± SEM. ^*^p < 0.05, ^**^p < 0.01, and ^***^p < 0.001 compared to vehicle WT mice. ^#^*p* < 0.05, ^##^*p* < 0.01, and ^###^*p* < 0.001 compared to LD EtOH diet WT mice.

The objective of this study was to ascertain whether mice that developed alcohol-induced fatty liver syndrome could recuperate through the administration of MF001. To this end, an LD EtOH diet was provided to the mice for 4 weeks, as illustrated in Fig 6A. In the final four-week period, various doses of MF001 (0.1, 0.2, and 2 g/kg) were administered concurrently during the recovery process. No differences in liver weights were observed between the groups (Fig 6B) and no significant differences in body weight, fat, lean mass, and food consumption (Fig 6C). To validate the histological results, H&E staining was performed, which revealed an increase in lipid droplets in the Con group and a decrease in the MF001-treated group (Fig 6D). Serum TG and TC levels were in accordance with the H&E staining data (Fig 6E and 6F). In addition, the levels of ALT and AST were lower in MF001-treated mice than in the Con mice (Fig 6G and 6H). It is noteworthy that NEFA expression was suppressed in the MF001-treated group compared to that in the Con group following the induction of fatty liver syndrome (Fig 6I). Furthermore, acetaldehyde expression markedly increased in response to MF001 treatment (Fig 6J). Furthermore, we sought to ascertain whether MF001 ameliorates fatty liver disease in mice with alcohol-induced fatty liver disease. To confirm this, the expression levels of lipogenic genes were measured, and it was observed that the expression levels of lipogenic genes (*Srebp-1c*, *Fasn*, *Acc1*, *Scd1*, and *Red*) were regulated by MF001 (Fig 7A and 7B), similar to the results of the experiment that confirmed the effect of MF001 on alcohol-induced fatty liver syndrome. The levels of lipogenic proteins (FASN, ACC1, and SCD1) were elevated in Con mice fed an LD EtOH diet and were reduced in MF001-treated mice (Fig 7C and 7D). MF001 treatment reduced the fatty acid oxidizing genes, *Pparα*, *Pgc-1α*, *Cpt-1α*, and *Cpt-1β* in alcohol induced mice liver in a dose dependent manner (S3A Fig in S1 File). The concentration of MDA and the levels of γ-GTP were reduced in mice treated with MF001 compared to those treated with the Con (Fig 7E and 7F). Based on these results, we can confirm that alcohol-induced fatty liver syndrome was reversed by MF001 and that recovery from inflammatory reactions is a possibility.

**Fig 8:**
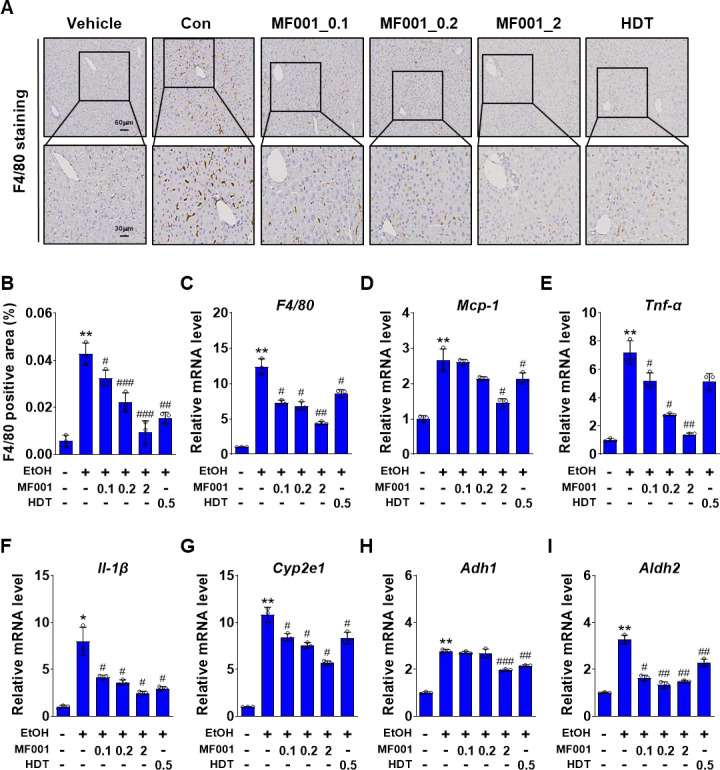
MF001 relieved alcohol-induced inflammation and fatty liver symptoms in mice. (A) Representative images of liver sections stained with F4/80 (scale bars: 60 μm and 30 μm) and (B) quantitative graphical representation. (C-F) qPCR analysis of *F4/80*, *Mcp-1*, *Tnf-α*, and *Il-1β* mRNA expressions in these samples. (G-I) *Cyp2e1*, *Adh1*, and *Aldh2* mRNA levels were measured using qPCR. Values represent the mean ± SEM. ^**^p < 0.01, and ^***^p < 0.001 compared to vehicle WT mice. ^#^*p* < 0.05, ^##^*p* < 0.01, and ^###^*p* < 0.001 compared to LD EtOH diet WT mice.

Histological analysis revealed that F4/80 staining for inflammation was elevated in the Con mice compared to the vehicle-treated mice, and this effect was reversed in the MF001-treated mice (Fig 8A and 8B). Furthermore, MF001 treatment resulted in reduced *F4/80* gene expression (Fig 8C). The expression levels of several inflammation-related genes, including *Mcp-1*, *Tnf-α*, and *Il-1β*, were decreased by MF001. Serum levels of TNF-α and IL-1β also significantly reduced with the MF001 treated group (Fig 8D–F and S3B Fig in S1 File). In addition, not only the expression levels of genes involved in acetaldehyde production and EtOH metabolism, *Cyp2e1* and *Adh1*, were reduced by MF001 (Fig 8G–I), but also the serum ALDH2 level showed reduced activity (S3C Fig in S1 File). In conclusion, MF001 exerts anti-inflammatory effects by modulating the immune response, thereby protecting against alcohol-induced fatty liver syndrome and improving the condition of patients with this disease (S4 Fig in S1 File). In conclusion, MF001 has the potential to be developed into a functional material with efficacy against alcohol-induced liver dysfunction.

## Discussion

Investigating the roles of various enzymes in alcohol metabolism is crucial for developing strategies to prevent alcohol-induced fatty liver disease. In addition, it is important to consider complimentary interventions, such as organic edible compounds, which not only provide protective effects but also facilitate recovery from alcohol-induced liver damage. Accordingly, this study examined the functional attributes of a yeast-derived compound, MF001, which exhibited aldehyde-reducing properties and mitigated the deleterious consequences of alcohol consumption, including the alleviation of hepatic lipid accumulation and inflammation.

For several centuries, herbal remedies, including extracts from medicinal plants and fruits rich in polyphenolic properties, vitamins, and antioxidants that scavenge free radicals, have been used as therapeutics for alcohol abuse and hangover [14,31]. This was because of the assumption that oxidative stress is a primary mediator of hangover syndrome and that various antioxidants can mitigate the effects of alcohol exposure [32]. Nevertheless, contemporary research and medical practice have identified more effective strategies to address this issue, including the use of extract lysates derived from a yeast strain as a potential solution. ARC generated from both ALDH and GSH demonstrated high efficacy in providing relief from the symptoms of hangover in South Asians [17]. Nevertheless, there are limited reports and comprehensive studies on this compound, particularly those conducted with proper *in vivo* experiments. The current study confirmed and validated the efficacy of ARC-MF001 as a therapeutic agent for alcohol-induced fatty liver disease.

A previous study reported the absence of an evident correlation between blood acetaldehyde levels and hangover severity in human subjects, thereby suggesting that acetaldehyde may only play a minor role in the adverse symptoms experienced during hangovers [33]. Consumption of EtOH can have a profound impact on inflammatory processes, with a complex relationship between EtOH exposure and immune responses [34]. The literature on this topic is inconclusive, with some studies reporting cytokine release during hangovers and adverse effects [35], whereas others have not found such results [35]. For example, one study found that intraperitoneal administration of 4 g/kg of EtOH to Sprague–Dawley rats resulted in a significant elevation of IL-6 and TNF-α levels in the liver and spleen [36]. These previous reports were used as a reference for testing whether the steady induction of EtOH via the LD EtOH diet for a period of three weeks at a lower dose in mouse models could result in the release of inflammatory cytokines. Furthermore, the administration of MF001 to these mouse models alleviated alcohol-induced inflammation.

MF001, which plays a significant role in alcohol metabolism, was identified as an anti-aldehyde compound in an earlier study [17]. Oxidation of alcohol to acetaldehyde via ADH and CYP2E1 is regarded as the rate-limiting step in alcohol metabolism [37]. During excessive alcohol consumption, ADH detoxifies saturated alcohol and induces the enzyme CYP2E1, leading to the generation of ROS and superoxide, causing damage to the liver and other organs [38]. Data from this study indicated that MF001 protects hepatocytes from ROS generation following alcohol induction. Despite the observed downregulation of ADH1 and CYP2E1 mRNA expression, ethanol still appears to be converted to acetaldehyde. In these results, a key question remains the mechanism by which ethanol is oxidized to acetaldehyde under high-dose MF-001 treatment. While MF001 increases ALDH2 and GST activity-enzymes responsible for detoxifying aldehydes-the mRNA expression levels of CYP2E1 and ADH1, the primary ethanol-oxidizing enzymes, are significantly reduced. This raises an important mechanistic gap: what enzyme is converting ethanol to acetaldehyde under these conditions. In the case of CYP2E1, substrate-induced stabilization of the protein is well-documented [39], and residual ADH1 protein may retain catalytic activity for some time. In addition, alternative ethanol-metabolizing enzymes such as catalase or other ADH isoforms like ADH2 and ADH3 may play compensatory roles under these conditions [40]. Catalase, in particular, can oxidize ethanol in the presence of hydrogen peroxide and may become more relevant under oxidative stress [41]. Although CYP2E1 activity was not directly assessed in this study, future studies are planned to explore its enzymatic function and upstream regulatory mechanism in more detail. The increased activities of ALDH2 and GST observed in MF001-treated tissues may reflect a compensatory response to elevated levels of acetaldehyde or reactive metabolites. If ADH1 and CYP2E1 enzymatic functions are indeed impaired, the origin of acetaldehyde could involve non-canonical mechanisms, such as non-enzymatic oxidation or lipid peroxidation byproducts [42]. Even though one limitation of this study was the lack of determination of the precise metabolic pathway involved in the mechanism of action of MF001, to clarify this metabolic paradox, further studies are needed to assess the actual protein levels and activities of ADH1, CYP2E1, and catalase, as well as to perform time-course measurements of ethanol and acetaldehyde concentrations under MF001 treatment.

## Conclusion

The yeast-derived aldehyde-reducing compound MF001 has been demonstrated to reduce lipid accumulation by decreasing lipid peroxidation, lipid metabolism, and inflammation in the liver, and rescuing the damaged liver from alcohol-induced fatty liver symptoms. MF001 has the potential to serve as a therapeutic compound that not only mitigates the adverse effects of excessive alcohol consumption, such as hangovers, but also protects the liver from the development of fatty liver syndrome.

## Supporting information

S1 File **S1 Fig.** MF001 reduces fatty acid oxidation and EtOH regulating genes. (A) Fatty acid oxidation gene expression levels of *Pparα*, *Pgc-1α*, *Cpt-1α*, and *Cpt-1β* determined using qPCR in EtOH-, PA-, and MF001-treated mouse primary hepatocytes. (B) Ethanol reducing gene expression levels of *Cyp2e1*, *Adh1*, and *Aldh2* in determined using qPCR in EtOH-, PA-, and MF001-treated mouse primary hepatocytes. (C) Enzyme activity levels of ALDH2 in primary hepatocytes determined by ELISA. Values represent the mean ± SEM. ^*^p < 0.05, ^**^p < 0.01, and ^***^p < 0.001 compared to mock primary hepatocytes. ^#^*p* < 0.05, ^##^*p* < 0.01, and ^###^*p* < 0.001 compared with EtOH- and PA-treated mouse primary hepatocytes. **S2 Fig.** MF001 reduces fatty acid oxidation and protects against alcohol-induced liver inflammation. (A) Expression of major fatty acid oxidation genes *Pparα*, *Pgc-1α*, *Cpt-1α*, and *Cpt-1β* determined using qPCR in mouse liver tissues. (B, C) Activity of inflammatory enzymes TNF-α and IL-1β and ethanol reducing enzyme ALDH2, determined using serum mouse samples by ELISA. Values represent the mean ± SEM. ^*^p < 0.05 and ^**^p < 0.01 compared to vehicle WT mice. ^#^*p* < 0.05 and ^##^*p* < 0.01 compared to LD EtOH diet WT mice. **S3 Fig.** MF001 attenuates alcohol-induced fatty acid oxidation and relieves alcohol-induced inflammation. (A) Expression of major fatty acid oxidation genes *Pparα*, *Pgc-1α*, *Cpt-1α*, and *Cpt-1β* alcohol induced and MF001 treated mouse liver tissues, determined using qPCR. (B, C) Activity of inflammatory enzymes TNF-α and IL-1β and ethanol reducing enzyme ALDH2, in serum of alcohol induced and MF001 treated mouse samples by ELISA. Values represent the mean ± SEM. ^*^p < 0.05, ^**^p < 0.01, and ^***^p < 0.001 compared to vehicle WT mice. ^#^*p* < 0.05 and ^##^*p* < 0.01 compared to LD EtOH diet WT mice. **S4 Fig.** Graphical Abstract. Schematic representation for the function of MF001 in liver. **S1 Table.** Primer sequences used for qPCR. **S1 Data.** Western blot raw image. **S2 Data.** Statistical analysis for One-way ANOVA.
(ZIP)

## Abbreviations

ACC1: acetyl-CoA carboxylase 1
ADH: alcohol dehydrogenase
ALDH: acetaldehyde dehydrogenase
ALT: alanine aminotransferase
ARC: , aldehyde-reducing composition
AST: aspartate aminotransferase
BSA: bovine serum albumin
Con: control
CYP2E1: cytochrome P450, family 2, subfamily e, polypeptide 1
EtOH: ethanol
FASN: fatty acid synthetase
FBS: fetal bovine serum
GRAS: generally recognized as safe
GSH: glutathione
HDT: Hovenia dulcis Thunb. extract powder
IL: interleukin
LD: Lieber-DeCarli
MCP-1: monocyte chemoattractant protein-1
MDA: malondialdehyde
NEFA: non-esterified fatty acid
ORO: oil red o
PA: palmitic acid
PBS: phosphate-buffered saline
qPCR: real-time quantitative polymerase chain reaction
ROS: reactive oxygen species
SCD1: stearoyl-CoA desaturase 1
SREBP: sterol-regulatory element binding protein
TC: total cholesterol
TG: triglyceride
TNF-α: tumor necrosis factor-α
WT: wild-type
γ-GTP: γ-glutamyl transferase
