# Supplementary material for: A novel yeast-derived aldehyde-reducing compound MF001 protects against alcohol-induced liver damage
Source: PLoS One. 2025 Jul 10;20(7):e0327648. doi: 10.1371/journal.pone.0327648 (PMC12244695; doi:10.1371/journal.pone.0327648)

Fig. 1B original western blot images

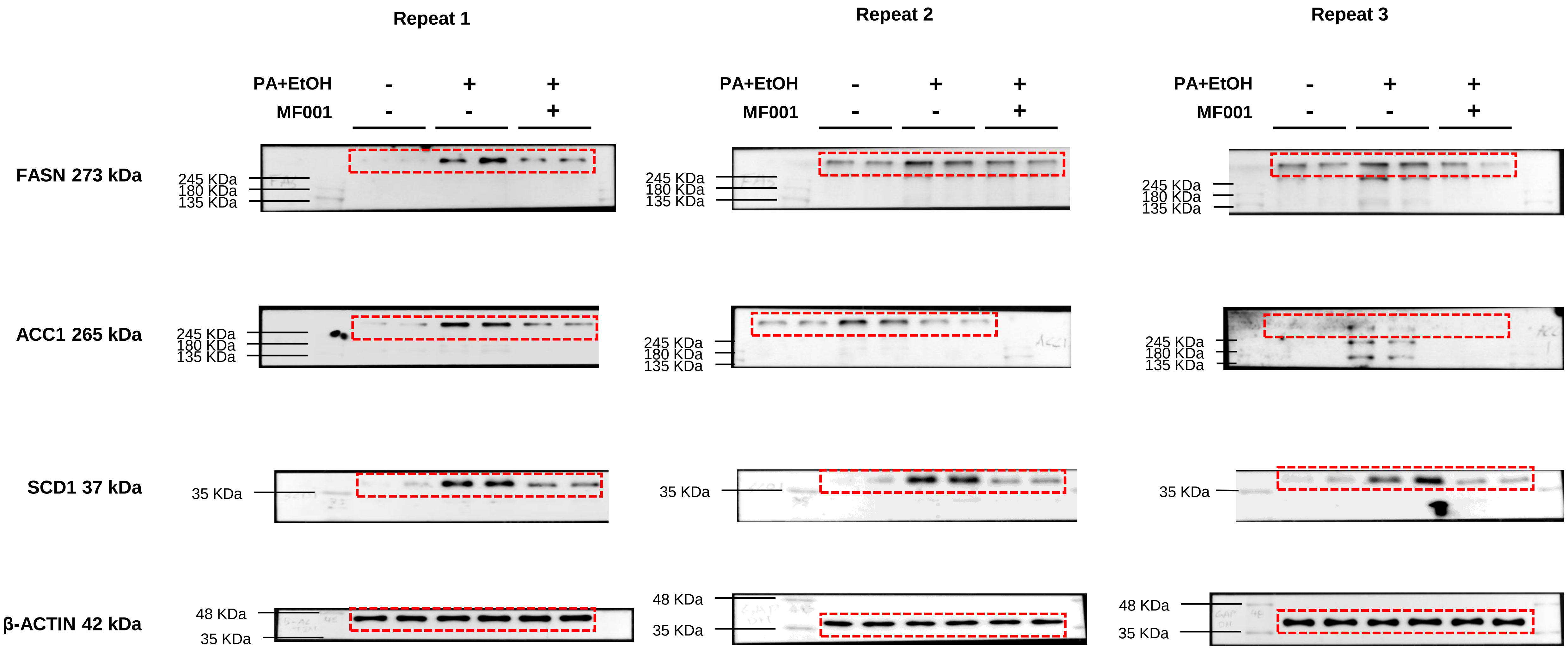

Fig. 4C original western blot images

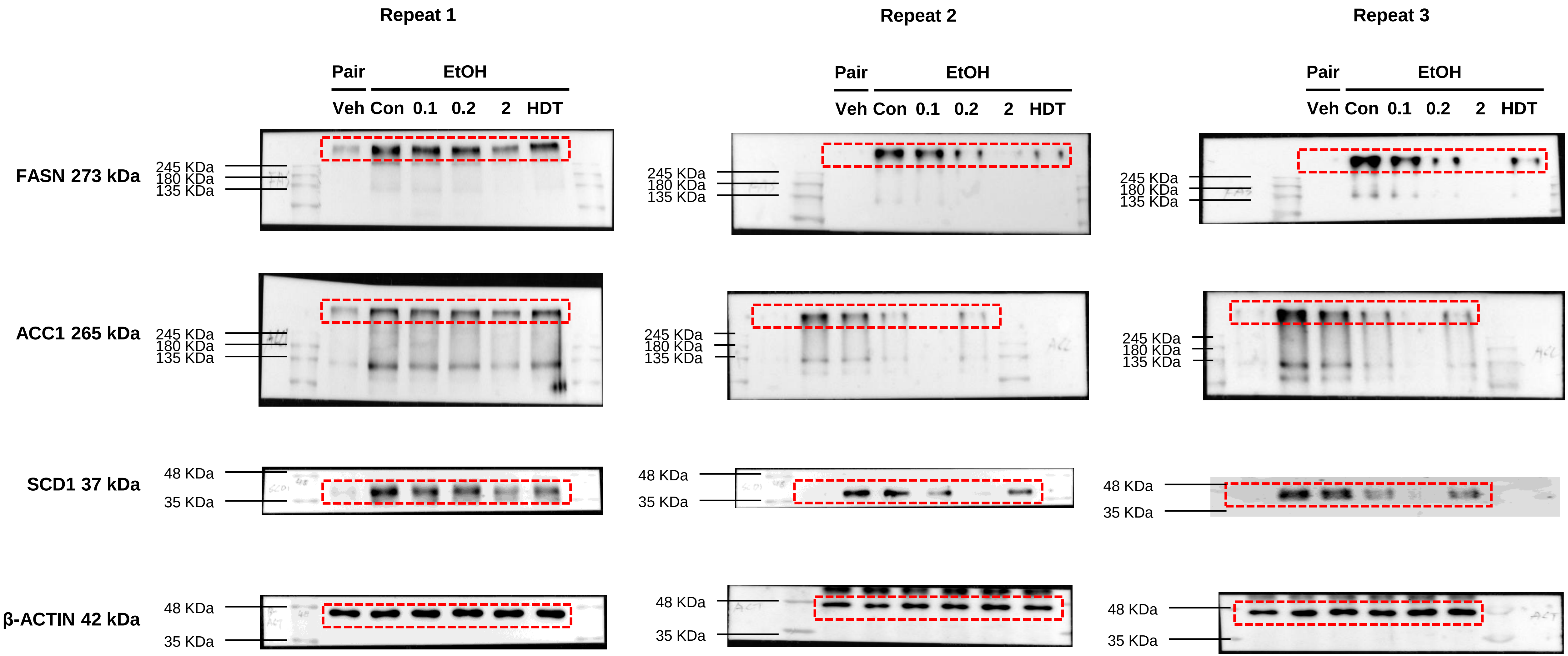

Fig. 7C original western blot images

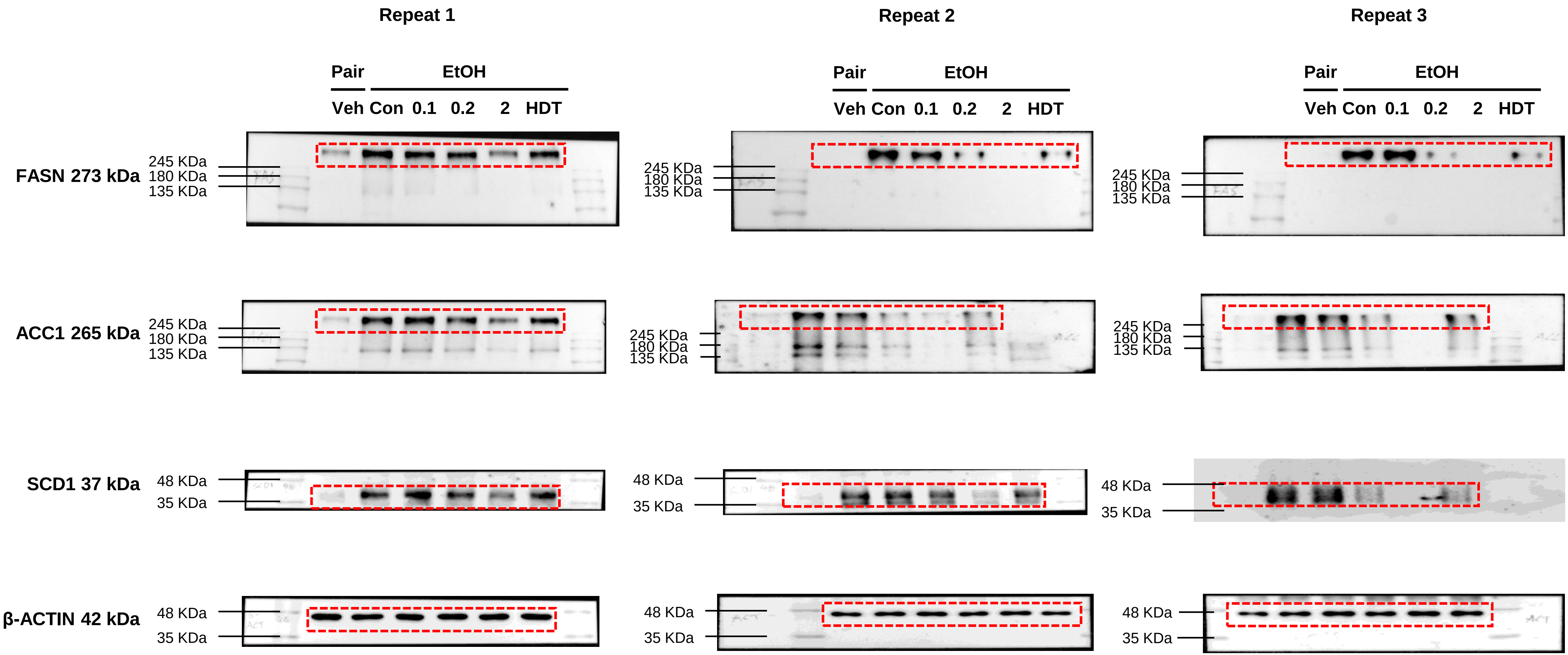

Supplement: S1 File — S1 Fig. MF001 reduces fatty acid oxidation and EtOH regulating genes. (A) Fatty acid oxidation gene expression levels of Pparα, Pgc-1α, Cpt-1α, and Cpt-1β determined using qPCR in EtOH-, PA-, and MF001-treated mouse primary hepatocytes. (B) Ethanol reducing gene expression levels of Cyp2e1, Adh1, and Aldh2 in determined using qPCR in EtOH-, PA-, and MF001-treated mouse primary hepatocytes. (C) Enzyme activity levels of ALDH2 in primary hepatocytes determined by ELISA. Values represent the mean ± SEM. *p < 0.05, **p < 0.01, and ***p < 0.001 compared to mock primary hepatocytes. #p < 0.05, ##p < 0.01, and ###p < 0.001 compared with EtOH- and PA-treated mouse primary hepatocytes. S2 Fig. MF001 reduces fatty acid oxidation and protects against alcohol-induced liver inflammation. (A) Expression of major fatty acid oxidation genes Pparα, Pgc-1α, Cpt-1α, and Cpt-1β determined using qPCR in mouse liver tissues. (B, C) Activity of inflammatory enzymes TNF-α and IL-1β and ethanol reducing enzyme ALDH2, determined using serum mouse samples by ELISA. Values represent the mean ± SEM. *p < 0.05 and **p < 0.01 compared to vehicle WT mice. #p < 0.05 and ##p < 0.01 compared to LD EtOH diet WT mice. S3 Fig. MF001 attenuates alcohol-induced fatty acid oxidation and relieves alcohol-induced inflammation. (A) Expression of major fatty acid oxidation genes Pparα, Pgc-1α, Cpt-1α, and Cpt-1β alcohol induced and MF001 treated mouse liver tissues, determined using qPCR. (B, C) Activity of inflammatory enzymes TNF-α and IL-1β and ethanol reducing enzyme ALDH2, in serum of alcohol induced and MF001 treated mouse samples by ELISA. Values represent the mean ± SEM. *p < 0.05, **p < 0.01, and ***p < 0.001 compared to vehicle WT mice. #p < 0.05 and ##p < 0.01 compared to LD EtOH diet WT mice. S4 Fig. Graphical Abstract. Schematic representation for the function of MF001 in liver. S1 Table. Primer sequences used for qPCR. S1 Data. Western blot raw image. S2 Data. Statistical analysis for One-way ANO [file pone.0327648.s001.zip › Supporting Information/S1 Data.pdf]
